# Early Changes in Nitrate Uptake and Assimilation Under Drought in Relation to Transpiration

**DOI:** 10.3389/fpls.2020.602065

**Published:** 2020-12-23

**Authors:** Vít Gloser, Michaela Dvorackova, Daniel Hernandez Mota, Bojana Petrovic, Patricia Gonzalez, Christoph Martin Geilfus

**Affiliations:** ^1^ Department of Experimental Biology, Faculty of Science, Masaryk University, Brno, Czechia; ^2^ Division of Controlled Environment Horticulture, Faculty of Life Sciences, Albrecht Daniel Thaer-Institute of Agricultural and Horticultural Sciences, Humboldt-University of Berlin, Berlin, Germany

**Keywords:** nitrate transport, nitrogen deficiency, nitrate reductase, pH, xylem, apoplast

## Abstract

Soil drying combined with nitrogen (N) deficiency poses a grave threat to agricultural crop production. The rate at which nitrate (NO_3_^−^) is taken up depends partly on the uptake and transpiration of water. Rapid changes in nitrate assimilation, in contrast to other N forms, may serve as a component of the plant stress response to drought because nitrate assimilation may lead to changes in xylem pH. The modulation of xylem sap pH may be relevant for stomata regulation *via* the delivery of abscisic acid (ABA) to guard cells. In several factorial experiments, we investigated the interactions between nitrate and water availability on nitrate fate in the plant, as well as their possible implications for the early drought-stress response. We monitored the short-term response (2–6 days) of nitrate in biomass, transport to shoot and reduction in *Pisum sativum*, *Hordeum vulgare*, *Vicia faba*, and *Nicotiana tabacum* and correlated this with sap pH and transpiration rates (TRs). Cultivation on inorganic substrate ensured control over nutrient and water supply and prevented nodulation in legume species. NO_3_^−^ content in biomass decreased in most of the species under drought indicating significant decline in NO_3_^−^ uptake. *Hordeum vulgare* had the highest NO_3_^−^ concentrations in all organs even under drought and low NO_3_^−^ treatment. This species can likely respond much better to the combined adverse effects of low NO_3_^−^ and water scarcity. Nitrate reductase activity (NRA) was reduced in both roots and leaves of water deficient (WD) plants in all species except *H. vulgare*, presumably due to its high NO_3_^−^ contents. Further, transient reduction in NO_3_^−^ availability had no effect on sap pH. Therefore, it seems unlikely that NRA shifts from shoot root leading to the supposed alkalization of sap. We also did not observe any interactive effects of NO_3_^−^ and water deficiency on transpiration. Hence, as long as leaf NO_3_^−^ content remains stable, NO_3_^−^ availability in soil is not linked to short-term modulation of transpiration.

## Introduction

Nitrate (NO_3_^−^) is a very important nitrogen (N) source for almost all plants, particularly crop plants. Fluctuations in NO_3_^−^ availability considerably alter both rates of NO_3_^−^ transport and assimilation (i.e., reduction) within the plant (Peuke et al., 1996; Lips, 1997). When external NO_3_^−^ availability decreases, its transport to the plant and to the shoot is typically reduced (Hachiya and Sakakibara, 2017). Low NO_3_^−^ availability can also lower the transpiration rate (TR; Wilkinson et al., 2007) and consequently affect water use by crops. Araus et al. (2020) showed that changes in NO_3_^−^ availability may lead to changes in the expression of drought-response genes; this response also involves the drought stress hormone abscisic acid (ABA). An interesting detail is that nitrogen supply to roots enhances stomatal opening, provided that plants are well watered (Wilkinson et al., 2007; Matimati et al., 2014). Conversely, nitrogen deficiency can cause rapid reduction of gas exchange (Chapin et al., 1988). This response can occur without any change in leaf water potential (WP); it is mediated by increased ABA production and a consequent decrease in gas exchange similar to the response to water deficiency (Chapin, 1991). The N status of the plant also influences gas exchange because N is involved in many processes related to photosynthesis (Lu and Zhang, 2000). The variation in the rate of carbon fixation and, consequently in leaf intercellular CO_2_ concentration, strongly affects stomatal aperture (Roelfsema et al., 2002, 2006). Additionally, the rate of ATP generation in the primary processes of photosynthesis is also one of the regulators of stomatal conductance (Tezara et al., 1999; Tominaga et al., 2001).

Thus, there seems to be a link between nitrogen (NO_3_^−^) availability and the response to drought. Moreover, decreasing water availability in soil can negatively affect NO_3_^−^ uptake and assimilation (Lawlor and Cornic, 2002; Azedo-Silva et al., 2004). Nevertheless, it is not completely clear whether these early changes in NO_3_^−^ metabolism can also serve as a component of the plant stress response system. For an overall summary of the interdependence of water and nitrogen use, the reader is referred to the recent review by Plett et al. (2020).

Experimental evidence also indicates that changes in NO_3_^−^ availability even within the non-deficient range may have a rapid regulatory effect on stomatal opening (Wilkinson et al., 2007). The underlying mechanism may involve changes in xylem sap pH, which is relevant for the compartmental distribution of ABA between mesophyll cells and the guard cell apoplast (Wilkinson et al., 1998; Davies et al., 2002; Geilfus et al., 2015). This ABA is likely being produced in the shoot (Dodd et al., 2009; McAdam et al., 2016; Li et al., 2018). For example, apoplastic alkalization during drought or other environmental stresses promotes ABA accumulation in the guard cell apoplast and triggers stomatal closure (Wilkinson and Davies, 2002; Geilfus et al., 2017). Notably, increasing NO_3_^−^ levels was also reported to increase leaf apoplast pH (Jia and Davies, 2007). This could be due to altered NO_3_^−^ assimilation, *via* altered NO_3_^−^ reductase activity (NRA; Wilkinson, 2004).

Nitrate assimilation is a relevant factor for apoplastic pH because of the number of electrons required to reduce NO_3_^−^ to NH_4_^+^. As a result, strongly basic hydroxides are formed, which are buffered by newly synthesized organic acids (i.e., malic acid) as otherwise the alkalizing hydroxides can drastically alter cytosolic pH (Dijkshoorn, 1962). Transporting organic acid anions (e.g., malate^2−^), into the apoplast is considered as the cause for the depletion of free apoplast protons. In the slightly acidified apoplast, free protons associate with the malate anion, thereby increasing the pH. Nonetheless, the relevance of drought for this apoplastic pH-modulating effect is still unclear. Drought is known to increase apoplastic pH, as shown for field bean (Karuppanapandian et al., 2017), sunflower (Gollan et al., 1992), asiatic dayflower (*Commelina communis*; Wilkinson and Davies, 1997), or maize (Goodger et al., 2005). Drought also has a significant effect on NRA in some species (Larsson et al., 1989; Azedo-Silva et al., 2004; Correia et al., 2005). Limited availability of NO_3_^−^ as substrate for NRA due to the reduced uptake and transport under drought is one possible explanation for the downregulation of NRA (Azedo-Silva et al., 2004). NRA may also decrease due to lower NR activation under drought (Brewitz et al., 1996; Foyer et al., 1998). Moreover, the response of NRA to water availability may differ between species that differ in the primary site of NO_3_^−^ reduction (root vs. leaves), but to our knowledge no data on this has been published so far.

Given that resource availability in the environment fluctuates greatly over time, rapid plant responses to any fluctuations may significantly impact productivity. The interrelation between water availability and both uptake and reduction of NO_3_^−^ could be relevant for transpiration by modulating xylem sap pH – a factor that is linked to controlling plant transpiration and gas exchange in general. Besides the role of NO_3_^−^ in plant metabolism, in contrast to other N forms available in the soil, is its uptake more tightly linked to water movement toward plant roots (Marschner, 1995). Any assessment of the hypothesis on the relevance of NO_3_^−^ for transpiration must consider species-specific differences in the ability to take up and reduce NO_3_^−^ under drought. Thus, four species were evaluated that contrast in their nitrogen uptake and assimilation: *Pisum sativum*, *Hordeum vulgare*, *Vicia faba*, and *Nicotiana tabacum*. Moreover, the differential ability of diverse species to change xylem sap pH under drought stress is also certainly relevant (Sharp and Davies, 2009; Gloser et al., 2016). In this paper, we describe a series of experiments where we investigated the interactions between NO_3_^−^ availability and water availability and their implication for the early drought-stress response. The possible effect of the dominant site of NO_3_^−^ assimilation inside the plant on its drought response is also discussed.

## Materials and Methods

### Plant Material and Cultivation

Four species were used in experiments: green pea (*P. sativum* cv. *Premium*), spring barley (*H. vulgare cv. Heris*), bean (*V. faba cv*. *Merkur*), and tobacco (*N. tabacum cv. GAT*). Species differ in the distribution of NRA between roots and leaves (Andrews, 1986), as well as in the changes of xylem sap composition under water deficiency (Gloser et al., 2016). Plants were cultivated with the inorganic substrate Profile Porous Ceramic (PPC, PROFILE Products LLC, Buffalo Grove, IL, United States). This was done to ensure full control of nutrient availability. In all experiments, plants were initially grown in a greenhouse with temperature and humidity control. Five days before the drying treatment started, plants were moved to a growth chamber with 16/8 h light/dark photoperiod and the following conditions: mean photosynthetic photon flux density 400 μmol m^−2^ s^−1^, average temperature of 22°C (light) and 20°C (dark), and relative humidity 60% (constant). The position of plants for different treatments in the chamber was randomized. Due to the supply of NO_3_^−^, there were no nodules on roots of the legumes *V. faba* and *P. sativum*.

Five days after moving the plants to the growth chamber (day 0), two different experiments were started. In experiment 1, 40 plants of each of the four species were separated into two treatments. Irrigation was stopped in the water deficient (WD) treatment for 10 days, whereas well-watered (WW) plants were irrigated daily to full water holding capacity of the substrate with nutrient solution containing: 1.21 mM Ca(NO_3_)_2_, 1.6 mM KNO_3_, 380 μM KH_2_PO_4_, 540 μM MgSO_4_, 4 μM MnSO_4_, 1.7 μM ZnSO_4_, 0.3 μM CuSO_4_, 40 μM, H_3_BO_3_, 0.5 μM Na_2_MoO_4_, and 81 μM FeNa-EDTA (4 mM NO_3_^−^ N in total). Experiment 2 was a two-factorial experiment with water and nitrogen availability as factors. About 60 plants of *V. faba* and *P. sativum* were divided into the same two treatments as experiment 1 at day 0, i.e., in 30 WW and 30 WD plants. The 30 plants of each water treatment were then separated into two group’s á 15 plants. At day 0, in order to wash out the remaining nitrogen, containers of the plants that were supposed to experience lack of nitrogen were flushed with a large amount of nitrogen solution containing: 60.3 μM Ca(NO_3_)_2_, 79.5 μM KNO_3_, 750 μM K_2_SO_4_, 1.14 mM CaCl_2_, 380 μM KH_2_PO_4_, 540 μM, MgSO_4_, 4 μM MnSO_4_, 1.7 μM ZnSO_4_, 0.3 μM CuSO_4_, 40 μM, H_3_BO_3_, 0.5 μM Na_2_MoO_4_, and 81 μM FeNa-EDTA [0.2 mM NO_3_^−^ N in total; plants from this experimental group are named “low nitrogen (LN) plants”]. The missing NO_3_^−^ in LN solution was replaced with sulphate to retain both ionic and osmotic parity between treatments. Plants in “full nitrogen (FN) treatment” were watered with the initial solution described above. Model calculations based on solution volume retained (or additionally supplied) in each container, and composition of the solution showed large differences in total nitrate potentially available to plants during the experimental period when drying was applied, particularly between HN and LN treatments: WWHN: 3.1 mmol, WDHN 1.7 mmol, WWLN: 0.15 mmol, and WDLN: 0.08 mmol. Plant response was monitored regularly for up to 10 days after the start of treatments, making three harvests every 2–3 days.

### Measurements of Soil and Plant Water Relations

Substrate water content (SWC) was monitored using a ThetaProbe connected to a HH2 meter (Delta-T Devices, Cambridge, United Kingdom). SWC was also measured in each pot immediately prior to harvesting. The relationship between substrate WP and SWC is shown in Supplementary Figure S1 (Supplementary Material). Whole plant TR was determined gravimetrically prior to xylem sap collection. TR was measured at least for 1 h under normal cultivation conditions, and plant containers were covered with a plastic bag to prevent evaporation from the substrate. Plant leaf area was determined at the end of sampling using a scanner and the image analysis software ImageJ (NIMH, Bethesda, MD, United States). WP of the plant was measured either on young fully developed leaves or on the top part of the shoot (depending on the species), using the pressure chamber method before xylem sap sampling (see below).

### Xylem Sap Collection and Analysis

The collection of xylem sap and the measurement of leaf/stem WP (Boyer, 1967) and xylem sap pH were conducted between 2 and 7 h from photoperiod start. Xylem sap was collected either from leaf (*N. tabacum*) or stems (*P. sativum*, *H. vulgare*, and *V. faba*) using a pressure chamber (PWSC 3005, Soilmoisture Equipment Corp., Santa Barbara, CA, United States). Sap was sampled from the second or third fully developed leaf of *N. tabacum*. After cutting, the cut surface of the petiole/stem was rinsed with distilled water to remove contaminants from the cut cells and the petiole/stem was sealed in the pressure chamber. The first drop of xylem sap was discarded and the pressure was noted as the WP. Pressure was gradually increased up to approx. Around 0.2 MPa above the balancing pressure and 10–40 μl of xylem sap was collected from each leaf (or shoot) over approximately 10 min. The pH of xylem sap was taken by a pH microelectrode (Microelectrodes Inc., Bedford, MA, United States) connected to an MP220 pH meter (Mettler Toledo, Switzerland). Samples were stored at −20°C immediately after pH measurement, until further analysis.

### Determination of Nitrate in Biomass and Sap, Calculation of Delivery Rates

Nitrate was extracted from 50 mg of fresh biomass in 1 ml of hot (95°C) DI water for 30 min. NO_3_^−^ concentration in the extract as well as in the xylem sap samples was consequently assayed by a spectrophotometric method (Cataldo et al., 1975). The delivery rate of NO_3_^−^ was calculated as sap NO_3_^−^ concentration multiplied by water flux to leaves (g h^−1^) derived from the gravimetric TR measurement of the respective plant shortly before sap sampling.

### Nitrate Reductase Activity

Nitrate reductase activity was estimated in leaves and roots with an *in vivo* assay (Black et al., 2002). Four subsamples of leaf disks (~100 mg) or finely chopped roots (~200 mg) were combined with 5 ml of assay buffer (200 mol m^−3^ KNO_3_ and 5% propanol in 100 mol m^−3^ potassium phosphate buffer, pH 7.5) in 20 ml glass vials. The vials were closed and placed in the dark at 25°C on a shaker. Two replicate vials for each sample were removed from the shaker after 10 and 90 min and placed in boiling water for 15 min. The vials were cooled to room temperature and aliquots of assay buffer were collected and stored at −20°C. To determine nitrite concentration, 500 ml of 1% sulphanilamide in 3 M HCl and 500 ml of 0.02% N-naphthyl-ethylene-diamine hydrochloride in water were added to the thawed samples and kept in the dark at room temperature for 20 min, and the absorbance measured at 540 nm. Enzyme activity was calculated by comparing the amount of nitrite produced after 90-min incubation with that detected after 10 min (Black et al., 2002). We took the mean of two replicates and expressed NRA as μmol nitrite produced g (Fresh Mass)^−1^ (fine roots or leaves) h^−1^.

### Statistical Evaluation

The effect of drought was tested on WD and WW control plants. However, the different species and the various treatments differed in drying rates. To enable meaningful comparison across treatments and species, we used information about SWC and TR of each plant shortly before harvest. Plants were grouped such that both SWC and TR of WD plants were significantly lower than those of WW plants. WD treatment typically included plants with SWC 0.6 g g^−1^ and lower. Statistical analyses were performed using STATISTICA 12 (TIBCO Software Inc., Palo Alto, CA, United States). In the first group of results − comparison of the response to drought under full NO_3_^−^ supply − significant differences between means WW and WD treatments were tested by a *t*-test separately for species and parameter. In experiments with a two-factorial design, the combined effects of drought and N-deficiency were tested by a two-way ANOVA for each species separately. Normality of data and homogeneity of variances were tested prior to analysis and non-homogeneous datasets were log transformed. Spearman’s correlation analysis was used to evaluate the significance of correlations between the measured parameters. Correlation analyses included data of all harvested plants of each species.

## Results

### Effect of Water Availability on Plant Water Relations

A fall in water availability to approximately 50–60% of the maximum substrate water holding capacity (SWC = 1.1 g g^−1^) led to a significant decrease in the TR and the leaf WP in all examined species (Table 1). The decrease was particularly pronounced (approximately 50%) in *N. tabacum* and *P. sativum*. *Nicotiana tabacum* showed a significant decrease in WP with the value being three times lower relative to WW-conditions.

**Table 1:** Mean soil water content (SWC), whole plant TR and the leaf WP of plants with full water supply (well-watered; WW) and plant exposed to gradual drying of substrate for several days (water-deficient; WD).

Species	Treatment	SWC (g g^−1^)	TR (g m^−2^h^−1^)	WP (MPa)
*Vicia*	WW	0.86 ± 0.2 a	159.15 ± 8.4 a	−0.24 ± 0.04 a
*faba*	WD	0.49 ± 0.2 b	121.80 ± 10.51 b	−0.41 ± 0.05 b
*Nicotiana*	WW	0.84 ± 0.02 a	76.5 ± 7.7 a	−0.20 ± 0.04 a
*tabacum*	WD	0.49 ± 0.04 b	35.8 ± 8.1 b	−0.65 ± 0.05 b
*Pisum*	WW	1.01 ± 0.01 a	263.72 ± 6.3 a	−0.52 ± 0.02 a
*sativum*	WD	0,43 ± 0.1 b	152.50 ± 18.5 b	−0.75 ± 0.09 b
*Hordeum*	WW	0.92 ± 0.04 a	146.9 ± 14.3 a	−0.54 ± 0.05 a
*vulgare*	WD	0.42 ± 0.1 b	96.6 ± 14.2 b	−0.70 ± 0.05 b

Presented are means and SEs of *Vicia faba* (*n* = 13), *Nicotiana tabacum* (*n* = 17), *P. sativum* (*n* = 31), and *H. vulgare* (*n* = 13). Significant differences (*p* ≤ 0.05) between treatments were tested separately for each species and are marked by different letters.

### Effect of Water Availability on NO_3_^−^ Content

Water deficient treatment significantly reduced NO_3_^−^ content in roots and leaves in three of the examined species and they also differed considerably in their response (Table 2). In *V. faba*, *N. tabacum* and *P. sativum*, NO_3_^−^ content was reduced both in leaves and roots in response to WD treatment (Table 2), but leaf NO_3_^−^ content was not significantly affected in *H. vulgare*. The concentration of NO_3_^−^ in xylem sap was also reduced in all species except *H. vulgare* (Table 2). The calculated delivery rates of NO_3_^−^ to leaves were significantly reduced (by 40–80%) in all species (Table 2). NRA significantly declined in WD treatment (Table 2) but the magnitude of the response was different among the different species. NRA in roots was greatly reduced (by 35–75%) in all species. NRA in leaves decreased in *V. faba*, *P. sativum*, and *N. tabacum* but not in *H. vulgare* (Table 2).

**Table 2:** Mean NO3^−^ content in the biomass of leaves ([NO3^−^]L) and roots ([NO3^−^]R), NO3^−^ concentration in the xylem sap ([NO3^−^]S) and the delivery rate of NO3^−^ to the shoot (DR); NRA in leaves (NRAL) and roots (NRAR) and the ratio of NRA in leaves and roots (NRAR/L), pH of the xylem sap (pHSAP).

Species	Treatment	[NO_3_^−^]_L_ (μmol g^−1^)	[NO_3_^−^]_R_ (*μ*mol g^−1^)	[NO_3_^−^]_S_ (μmol g^−1^)	DR (μmol h^−1^)	pH_SAP_	NRA_L_ (nmol g^−1^h^−1^)	NRA_R_ (nmol g^−1^h^−1^)	NRA_R/L_
*Vicia*	WW	10.31 ± 1.2 a	21.45 ± 3.8 a	3.49 ± 0.4 a	7.23 ± 1.0 a	5.95 ± 0.1 a	158.27 ± 21 a	227.90 ± 33.5 a	1.44
*faba*	WD	6.21 ± 0.8 b	10.10 ± 1.7 b	1.59 ± 0.4 b	1.37 ± 0.6 b	5.64 ± 0.1 b	85.47 ± 17 b	84.47 ± 17.2 b	0.99
*Nicotiana*	WW	5.66 ± 0.9 a	10.25 ± 1.1 a	9.09 ± 1.4 a	12.23 ± 1.5 a	6.96 ± 0.1 a	206.15 ± 34 a	49.29 ± 6.1 a	0.24
*tabacum*	WD	3.75 ± 0.4 b	5.65 ± 0.7 b	2.14 ± 0.9 b	2.83 ± 1.2 b	6.89 ± 0.1 a	147.89 ± 33 b	29.36 ± 3.8 b	0.20
*Pisum*	WW	5.08 ± 0.3 a	11.21 ± 0.9 a	2.14 ± 0.2 a	3.69 ± 0.5 a	6.07 ± 0.1 a	319.29 ± 35 a	376.51 ± 55.6 a	1.18
*sativum*	WD	3.95 ± 0.3 b	4.80 ± 0.7 b	1.05 ± 0.2 b	2.44 ± 0.8 b	5.84 ± 0.1 b	190.93 ± 24 b	125.96 ± 27.7 b	0.66
*Hordeum*	WW	93.06 ± 1.0 a	78.92 ± 11 a	7.87 ± 0.8 a	19.40 ± 1.7 a	6.26 ± 0.1 a	554.88 ± 66 a	324.18 ± 61 a	0.58
*vulgare*	WD	98.04 ± 6.8 a	82.47 ± 10 a	5.81 ± 0.9 a	12.17 ± 2.1 b	6.33 ± 0.1 a	655.02 ± 61 a	305.56 ± 56 a	0.47

Plants fully supplied with water (WW) or exposed to gradual drying of substrate for several days (WD). The table shows means ± SE for *V. faba* (*n* = 13), *N. tabacum* (*n* = 17), *P. sativum* (*n* = 31), and *H. vulgare* (*n* = 13). Significant differences (*p* = 0.05) between treatments were tested separately for each species and are marked by different letters.

### Impact of Nitrogen Deficiency Under Drought

**Figure 1:**
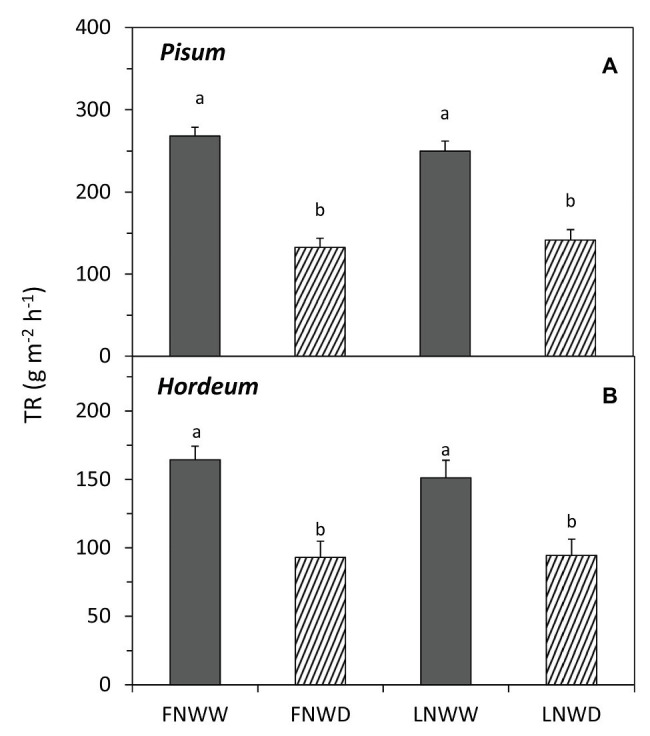
Mean transpiration rates (TRs) of *Pisum sativum* **(A)** and *Hordeum vulgare* **(B)** plants after 2–6 days treatment with different water and nitrogen availability. FNWW plants received 4 mM nitrate (NO3^−^) daily, FNWD plants received 4 mM NO3^−^ at the start but no water during the sampling period, LNWW plants received 0.2 mM NO3^−^ daily, and LNWD plants received 0.2 mM NO3^−^ at the start but no water during the sampling period. The graph shows means ± SE (*n* = 6–17). Significant differences (*p* ≤ 0.05) between treatments were tested separately for each species and are marked by different letters. FN, full nitrogen; LN, low nitrogen; WW, well-watered; and WD, water deficient.

**Figure 2:**
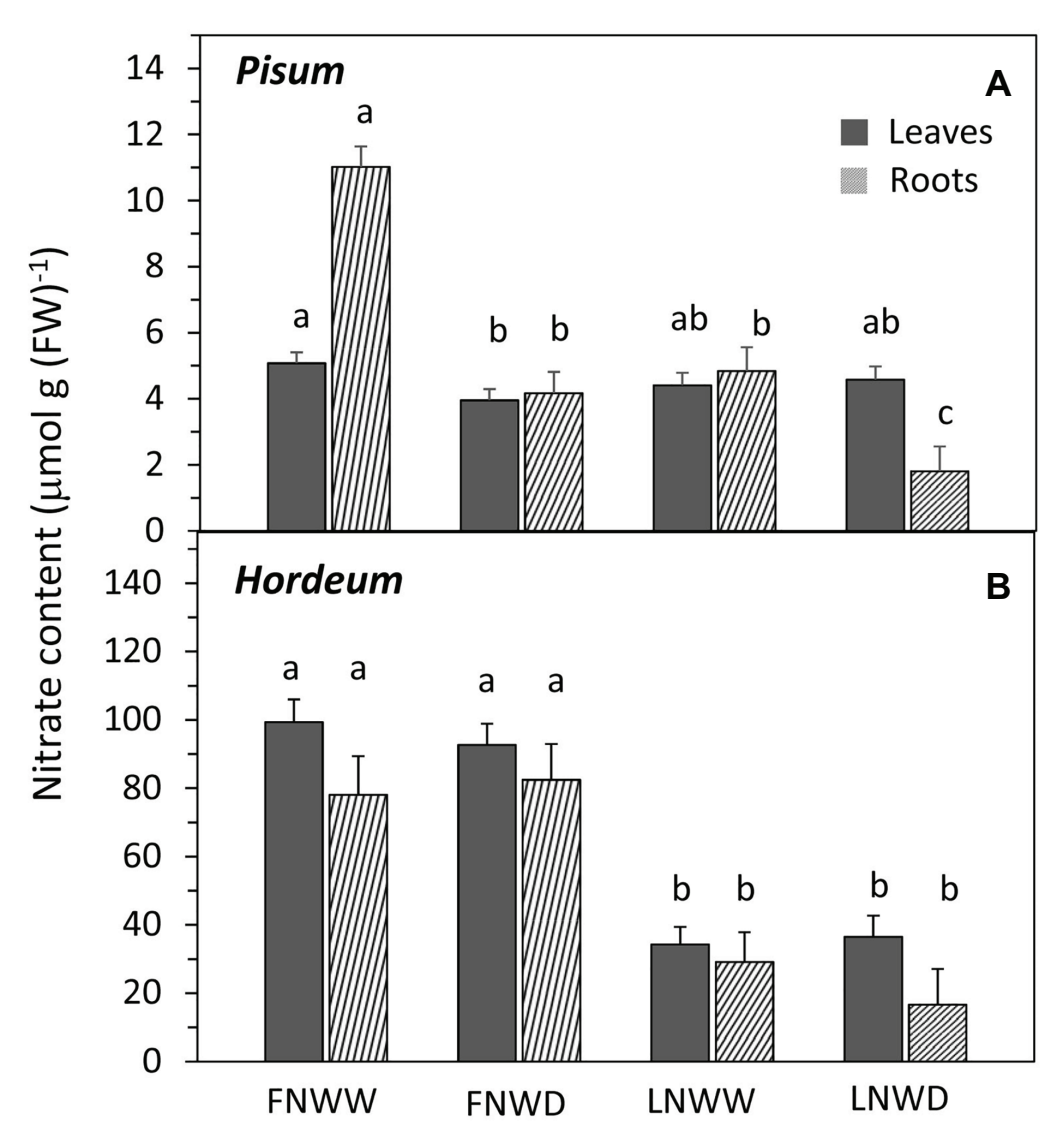
Mean NO3^−^ content in leaf and root biomass of *P. sativum* **(A)** and *H. vulgare* **(B)** plants after 2–6 days treatment with different water and nitrogen availability. FNWW plants received 4 mM NO3^−^ daily, FNWD plants received 4 mM NO3^−^ at the start but no water during the sampling period, LNWW plants received 0.2 mM NO3^−^ daily, and LNWD plants received 0.2 mM NO3^−^ at the start but no water during the sampling period. The graph shows means ± SE (*n* = 6–17). Significant differences (*p* ≤ 0.05) between treatments were tested separately for each organ of respective species separately and are marked by different letters. FN, full nitrogen; LN, low nitrogen; WW, well-watered; and WD, water deficient.

**Figure 3:**
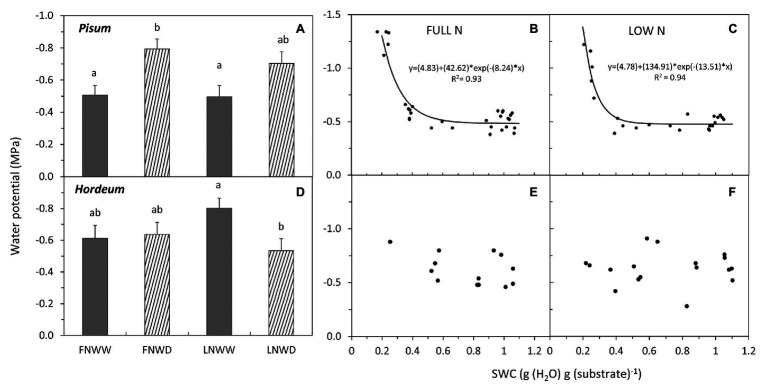
Mean water potential (WP) of *P. sativum* **(A,B,C)** shoots and *H. vulgare* **(D,E,F)** leaves after 2–6 days treatment with different water and nitrogen availability. FNWW plants received 4 mM NO3^−^ daily, FNWD plants received 4 mM NO3^−^ at the start but no water during the sampling period, LNWW plants received 0.2 mM NO3^−^ daily, and LNWD plants received 0.2 mM NO3^−^ at the start but no water during the sampling period. The graph **(A,D)** shows means in each treatment ± SE (n = 6–17). Significant differences (*p* ≤ 0.05) between treatments were tested separately for each species and are marked by different letters. Dynamic changes of WP with declining substrate water content (SWC) are shown in **(B,C)** for P. sativum; **(E,F)** for H. vulgare under full NO3^−^ (FN) and low NO3^−^ (LN) availability. Significant relationships are marked by a regression line with the corresponding equation. FN, full nitrogen; LN, low nitrogen; WW, well-watered; and WD, water deficient.

In *P. sativum* and *H. vulgare*, we studied the combined effects of water scarcity and lowered nitrogen availability in more detail. Contrary to expectations, reduction in NO_3_^−^ availability for several days had no negative effect on TR in either species (Figure 1; Table 3). Reduced NO_3_^−^ availability caused a significant reduction of NO_3_^−^ content in roots of both species but leaf content was not reduced in *P. sativum* (Figure 2; Table 3). The fall in TR under drought was similar under full or reduced NO_3_^−^ supply (Figure 1). WP of *P. sativum* decreased under water limitation, but in *H. vulgare* the decrease was not significant (Figure 3; Table 3).

**Table 3:** Significance of the experimental factors and their interaction was analyzed by two-way ANOVA.

Trait	*Pisum sativum*			*Hordeum vulgare*		
	Water	Nitrogen	W × N	Water	Nitrogen	W × N
TR	0.000	0.683	0.251	0.000	0.536	0.618
WP	0.000	0.460	0.550	0.115	0.561	0.059
[NO_3_^−^]_L_	0.197	0.960	0.082	0.718	0.000	0.472
[NO_3_^−^]_R_	0.000	0.000	0.854	0.698	0.000	0.420
NRA_L_	0.104	0.003	0.030	0.901	0.000	0.425
NRA_R_	0.000	0.000	0.021	0.396	0.027	0.603
[NO_3_^−^]_S_	0.000	0.002	0.676	0.179	0.000	0.781
Sap pH	0.004	0.870	0.953	0.201	0.714	0.439

TR, whole plant transpiration rate; WP, leaf water potential; [NO3^−^]L and [NO3^−^]R, NO3^−^ content in the biomass of leaves and roots; [NO3^−^]S, concentration of NO3^−^ in xylem sap; NRAL and NRAR, NO3^−^ reductase activity in leaves and roots; and sap pH, pH of the xylem sap.

**Figure 4:**
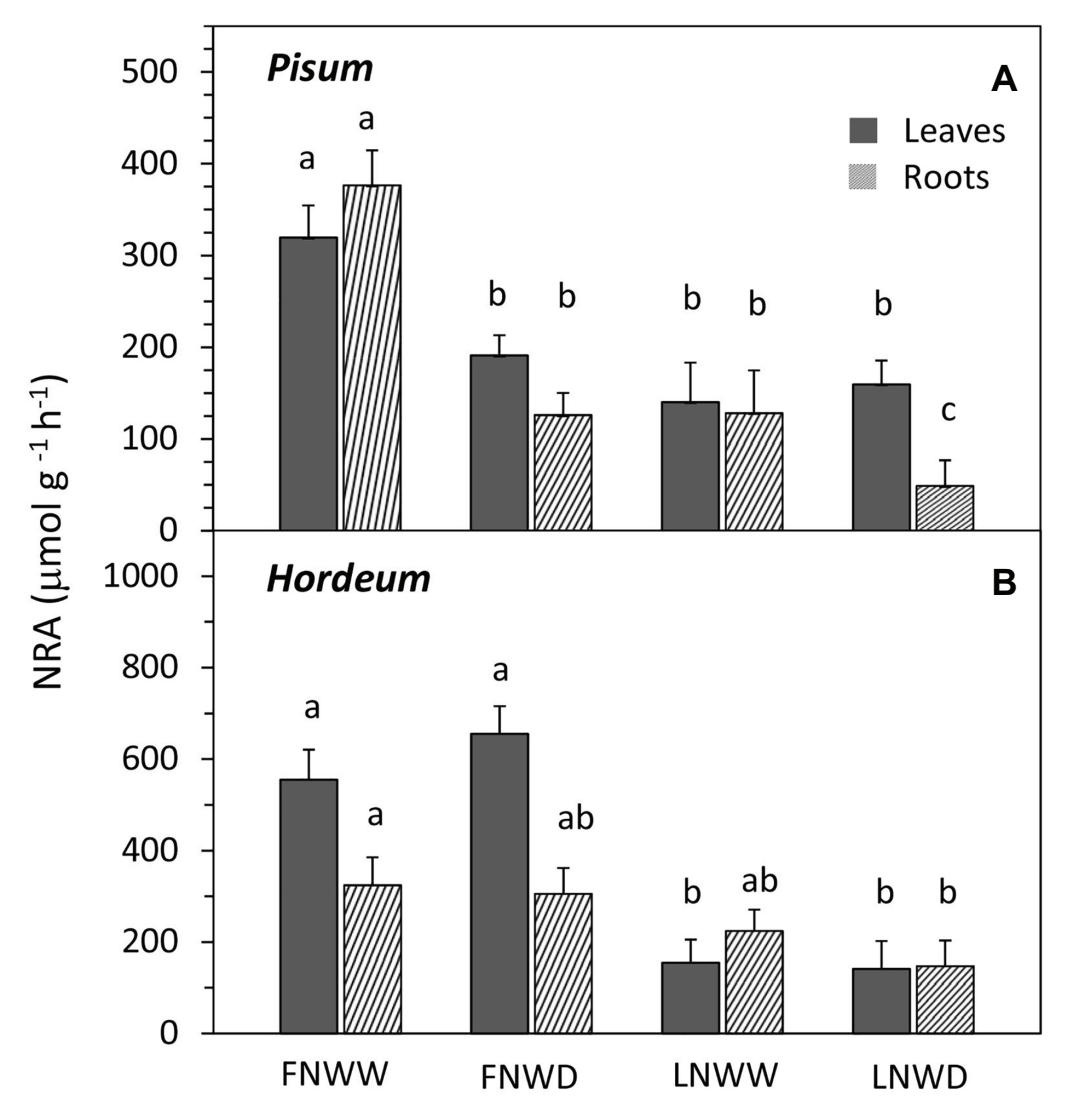
Mean nitrate reductase activity (NRA) in leaf and root biomass of *P. sativum* **(A)** and *H. vulgare* **(B)** plants after 2–6 days treatment with different water and nitrogen availability. FNWW plants received 4 mM NO3^−^ daily, FNWD plants received 4 mM NO3^−^ at the start but no water during the sampling period, LNWW plants received 0.2 mM NO3^−^ daily, and LNWD plants received 0.2 mM NO3^−^ at the start but no water during the sampling period. Presented are means ± SE (*n* = 6–17). Significant differences (*p* ≤ 0.05) between treatments were tested separately for each species and are marked by different letters. FN, full nitrogen; LN, low nitrogen; WW, well-watered; and WD, water deficient.

**Figure 5:**
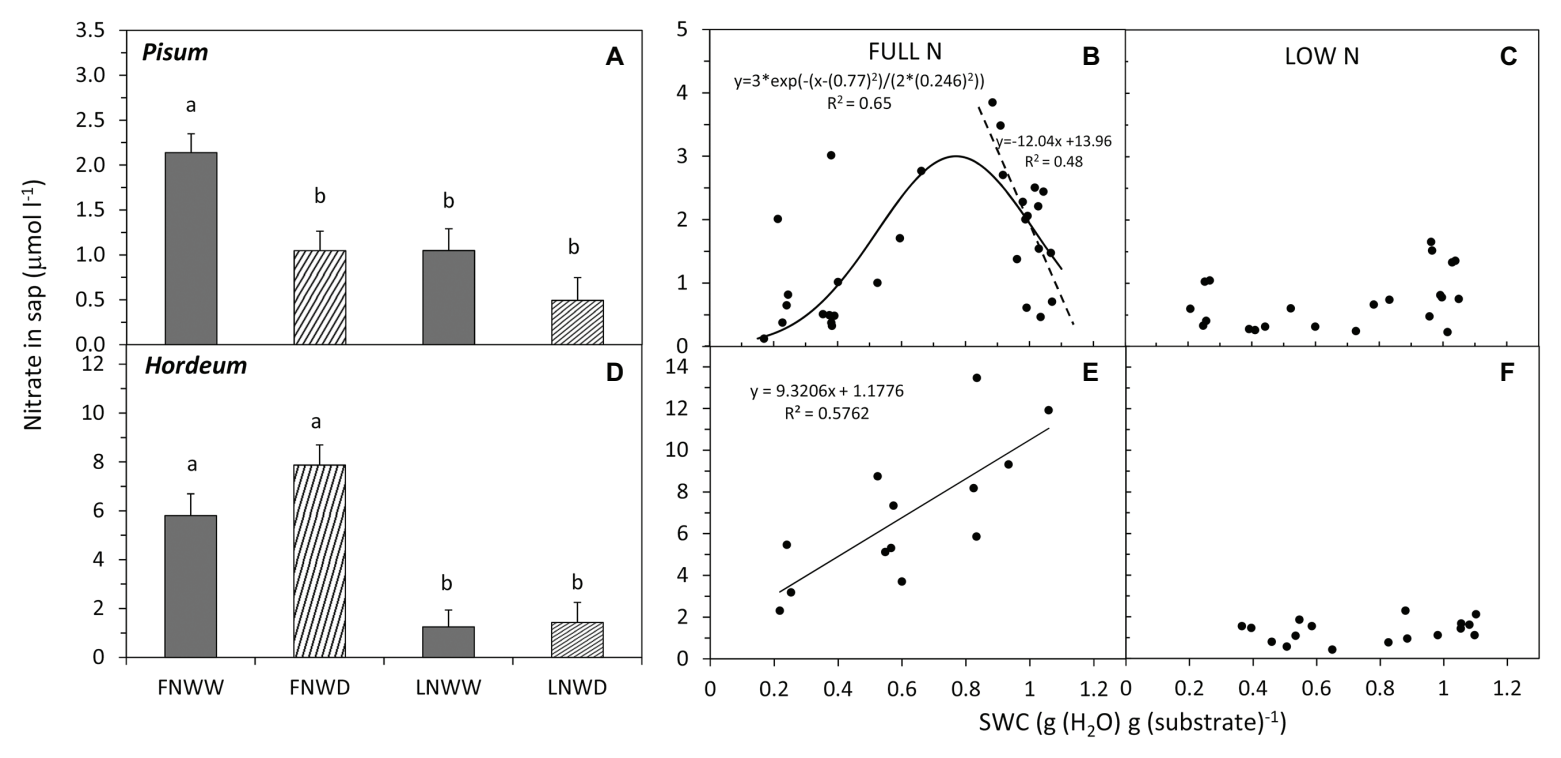
Mean NO3^−^ concentration in xylem sap of *P. sativum* **(A,B,C)** and *H. vulgare* **(D,E,F)** plants after 2–6 days treatment with different water and nitrogen availability. FNWW plants received 4 mM NO3^−^ daily, FNWD plants received 4 mM NO3^−^ at the start but no water during the sampling period, LNWW plants received 0.2 mM NO3^−^ daily, and LNWD plants received 0.2 mM NO3^−^ at the start but no water during the sampling period. The graphs **(A,D)** show means in each treatment ± SE (*n* = 6–17). Significant differences (*p* ≤ 0.05) between treatments were tested separately for each species and are marked by different letters. Dynamic changes of NO3^−^ concentration in sap with declining substrate water content (SWC) are shown in **(B,C)** for *P. sativum*; **(E,F)** for *H. vulgare* under full (FN) and low (LN) NO3^−^ availability. Significant relationships are marked by a regression line with the corresponding equation. In graph **(B)**, the separate linear regression line was fitted only to SWC above 0.7 g.g^−1^ (significant at *p* ≤ 0.003). FN, full nitrogen; LN, low nitrogen; WW, well-watered; and WD, water deficient.

Regression analysis revealed that the WP decrease in FN treatment occurred under higher SWC (Figure 3B) than in LN treated plants, where the decrease under low SWC was steeper (Figure 3C). Drought had a significant negative effect on NO_3_^−^ content in roots of *P. sativum* but no interaction of these factors was detected (Figure 2A, Table 3). NRA was significantly reduced in roots and leaves of both species in LN treatment (Figure 4). The negative effect of drought on NRA was observed only in *P. sativum* roots, and the negative effects of LN and reduced water availability were synergistic (Table 3), resulting in a much greater decrease of NRA of roots when both stress factors were applied simultaneously (Figure 4). Reduced NO_3_^−^ availability significantly reduced NO_3_^−^ transport in shoot xylem of both species (Figures 5A,D). From the group comparison, it is clear that the effect of drought was significant only under FN treatment in *P. sativum* (Figure 5A).

**Figure 6:**
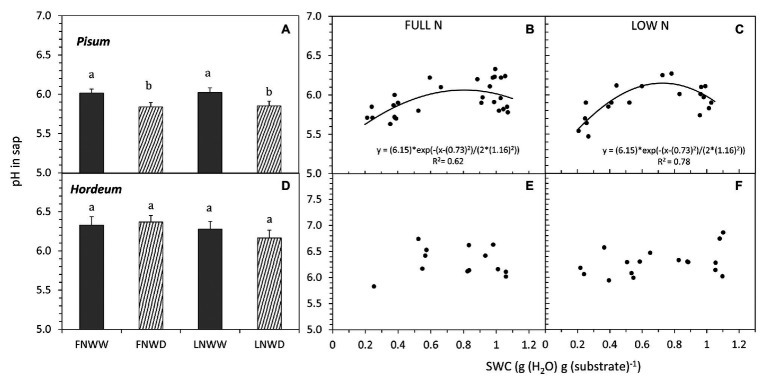
Mean pH in xylem sap (pH in sap) of *P. sativum* **(A,B,C)** and *H. vulgare* **(D,E,F)** plants after 2–6 days of treatments with different water and nitrogen availability. FNWW plants received 4 mM NO3^−^ daily, FNWD plants received 4 mM NO3^−^ at the start but no water during the sampling period, LNWW plants received 0.2 mM NO3^−^ daily, and LNWD plants received 0.2 mM NO3^−^ at the start but no water during the sampling period. The graphs **(A,D)** show means in each treatment ± SE (*n* = 6–17). Significant differences (*p* ≤ 0.05) between treatments were tested separately for each species and are marked by different letters. Dynamic changes of pH in sap with declining SWC are shown in **(B,C)** for *P. sativum*; **(E,F)** for *H. vulgare* under full (FN) and low (LN) NO3^−^ availability. Significant relationships are marked by a regression line with the corresponding equation. FN, full nitrogen; LN, low nitrogen; WW, well-watered; and WD, water deficient.

The relationship between changes in sap NO_3_^−^ concentration and SWC revealed two interesting findings. Firstly, under FN treatment, substrate drying led to a gradual decrease in NO_3_^−^ concentration in the sap, but this effect was not apparent in LN treated plants (Figures 5C,F). Secondly, in *P. sativum*, we observed a transient increase in NO_3_^−^ concentration in moderately dry substrate (approx. 0.7–0.8 g g^−1^ = 60–70% of the field water capacity, Figure 5B). The initial increase of sap NO_3_^−^ was confirmed also by a separate linear regression analysis of sap NO_3_^−^ concentrations when SWC was above 0.7 g g^−1^ (*p* ≥ 0.003). Xylem sap pH decreased in response to reduced water availability in *P. sativum* (Figure 6A; Table 3) but no significant response was detected in *H. vulgare* (Figure 6D; Table 3). Moreover, the relationship between pH and SWC showed a transient increase in *P. sativum* xylem sap pH, with this effect being apparent in both FN and LN treatments (Figures 6B,C). The range of SWC over which the pH increased was similar to that observed for the increase in sap NO_3_^−^ concentration. No such a response was observed in *H. vulgare* plants (Figures 6E,F).

## Discussion

### Water Availability and Nitrate Utilization by Plant

#### Drought Has a Negative Effect on NO_3_^−^ Availability and NO_3_^−^ Content

Under drought, the NO_3_^−^ content in biomass decreased in the majority of species we examined, implying a significant decline in NO_3_^−^ uptake (Table 2). This effect is probably due to a reduction of mass flow or diffusion-driven uptake of NO_3_^−^ from soil to root. It is known that short drought periods do not cause a decline in N-uptake due to low carbon (energy) supply from the shoot (Buljovcic and Engels, 2001). Decrease of SWC hinders the movement of soil solutions, due to increased hydraulic resistance as well as the reduced root surface area in contact with soil solution (Ehlers and Goss, 2016). Root shrinkage might also reduce the contact area (Nobel and Cui, 1992). The combined effect is decreased delivery of NO_3_^−^ to the root rhizodermis and its lowered uptake, unless the plant responds synthesizing new transport proteins, as suggested by Buljovcic and Engels (2001) and others (Bassett et al., 2014; Duan et al., 2016). Our results reveal a drought-induced reduction in NO_3_^−^-uptake, as indicated by changes in the NO_3_^−^ content in the biomass, for *V. faba*, *N. tabacum*, and *P. sativum* but not for *H. vulgare*. Barley had the highest NO_3_^−^ concentrations by far, and this did not change in response to drought. Moreover, it did not change much when only 0.2 mM NO_3_^−^ was supplied (Figure 2B). We do not know exactly why barley is unlike other species in this respect, but it is likely due to the result of selection by breeders who have attempted to increase nitrogen uptake efficiency for barley. These selection efforts have led to changes that combine favorable root morphology with more efficient NO_3_^−^ transporters (Bingham et al., 2012). Species with extensive root systems (e.g., Poaceae) typically show better NO_3_^−^ acquisition (Lainé et al., 1993).

We observed a transient but significant increase of NO_3_^−^ concentration in *P. sativum* xylem sap in the early phase of drying when fully supplied (FN) with NO_3_^−^ (Figure 5). No such increase was detected in plants supplied with LN. This suggests that this transient increase is linked to greater NO_3_^−^ uptake from substrate and cannot be explained only by NO_3_^−^ mobilization from root cell reserves. Moreover, evaporation of water from soil might potentiate short term NO_3_^−^ uptake by roots in as the soil concentration of NO_3_^−^ increases when water evaporates. Nevertheless, the contribution of this effect has never been properly evaluated. This mechanism could explain the previously observed transient increase in xylem NO_3_^−^ concentration following increased osmotic pressure of the solution (Larsson et al., 1989) or during the course of gradual drying (Wilkinson et al., 2007).

#### Drought Affects the Distribution of Nitrate in the Plant

Typically, higher NO_3_^−^ concentrations were observed in roots than in leaves of the species we examined (Table 2). Lower SWC led to a relatively greater reduction in NO_3_^−^ in roots than in leaves. The leaf NO_3_^−^ pool was significantly reduced in WD plants of all species except *H. vulgare*. Again, this might be attributable to better nitrogen uptake efficiency relative to other species. Field bean and pea are legumes that can fix atmospheric nitrogen (in our experiment, we used a rooting substrate that does not harbor the suitable N_2_-fixing symbiotic bacteria and no nodulation was observed). It is salient to note that legumes are not bred for higher abundances of root NO_3_^−^ transporters. Tobacco is a plant that is not primarily bred for the amount but rather the quality of its biomass. In tobacco, high contents of NO_3_^−^ or nitrile are undesirable because they are precursors of carcinogenic nitrosamines (Tso, 1972). In other words, breeding programs for nitrogen uptake efficiency are more common for barley and less relevant for the other crops. In their analysis of improvements in the nitrogen use efficiency of barley, Bingham et al. (2012) showed that over 75 years of breeding the nitrogen content of barley has increased as a result of both increased N uptake and utilization efficiencies. Barley might possess a more optimal composition of transporters for NO_3_^−^ uptake enabling enough nitrogen to be taken up when the available concentration of NO_3_^−^ is relatively low (0.2 mM), even in combination with reduced water availability. This is not only reflected as high NO_3_^−^ concentration in *H. vulgare* shoots but also as high sap concentrations in WD plants (Table 2). The first conclusion drawn by the results is that *H. vulgare*, in comparison to *P. sativum*, seems to maintain its capacity to take up NO_3_^−^ although NO_3_^−^ and water availability are low. It should be noted that under the water limited conditions applied in our experiments, *H. vulgare* did not show signs of severe drought stress such as a significantly lower WP (Figure 3). Also, it is well known that there are large differences in response to nitrogen availability and other environmental factors affecting productivity among plant cultivars (Giles et al., 2017). Therefore, the conclusions presented here may not be readily applicable to all varieties of examined species and this fact should be considered in future experiments.

#### Drought Negatively Affects Nitrate Assimilation

Many studies have demonstrated the strong negative effect of water deficit on NRA in leaves (Shaner and Boyer, 1976; Larsson et al., 1989; Foyer et al., 1998) and roots (Azedo-Silva et al., 2004). That water deficit has a more significant impact on NO_3_^−^ assimilation than on NO_3_^−^ uptake was suggested by Lawlor and Cornic (2002) as they observed a sharp decline in NRA under drought conditions, which was correlated with a reduction of relative water content. In our experiments, NRA was always reduced in roots and leaves of WD treated plants – with the salient exception of barley (Table 2). The major difference between barley and the other three species was that NO_3_^−^ content was not reduced by a water deficit. This clearly indicates that availability of the NR substrate (NO_3_^−^) is more relevant for NR activity as opposed to other drought-related regulatory signals. Shaner and Boyer (1976) showed that decline in transport of NO_3_^−^ to shoot can be more closely related to NRA than total content of NO_3_^−^ in leaf biomass of maize. In WD treatment of our experiments was NO_3_^−^ delivery to leaves also reduced. Possible “buffering effect” of high NO_3_^−^ in biomass and relatively smaller decrease in delivery rate in comparison to other examined species can be possible explanation of this different result. Besides NO_3_^−^ availability (Kaiser et al., 2002), the availability of carbohydrates (Larios et al., 2001), or products of nitrogen assimilation (Scheible et al., 1997) may have also been shown to have a regulatory effect on NRA. More detailed analysis of the relationship between NO_3_^−^ transport and NRA, including other regulatory signals, represents the next logical step in clarifying uncertainties related to the early impact of drought on nitrogen assimilation in barley.

### Nitrate Availability and Plant Response to Drought

#### Early Response to Drought and Nitrate Assimilation

In our experiments, we tested the hypothesis that NO_3_^−^ availability is not only affected by reduced water availability in the substrate but it can also modulate the plant drought response. Lips (1997) proposed a model explaining how NO_3_^−^ can be involved in the plant drought response. Declining water availability would reduce NO_3_^−^ uptake and transport to the shoot. This change then triggers a relatively larger decrease of NRA in the shoot rather than the root. Consequently, the increased proportion of NRA in roots would increase xylem sap pH due to the export of organic acids anions from the symplast into the apoplast (organic acid anions are formed during NO_3_^−^ reduction; see Introduction for details). Our experiments with four species indeed show that NO_3_^−^ concentration in organs and NO_3_^−^ transport to the shoot are mostly reduced even in the early phase of drought (Tables 2, 3), presumably due to restricted NO_3_^−^ uptake. On the other hand, under drought conditions, NRA was always more reduced in roots than in leaves. This was the case in all species that differed in the primary contribution of leaves and roots to plant NO_3_^−^ assimilation – legumes with relatively higher NRA in roots vs. non-legumes with rather equal distribution of NRA (Table 2). Therefore, the increased sap pH due to a shift of NRA from leaves to roots seems unlikely, just like the sequence of events that lead to pH-driven ABA accumulation as described above. In fact, we did not observe sap alkalization in any of the examined species when we compared only WW and WD treatments. On the contrary, *V. faba* and *P. sativum* responded to drought by sap acidification. Correlation analysis of data also did not find any relationship between NRA either in leaves or in roots and sap pH in *P. sativum*, *H. vulgare*, and *N. tabacum*. This result fits well with our previous multi-species study, which showed that the dominant site of NO_3_^−^ reduction has no effect on sap pH under drought (Gloser et al., 2016). On the other hand, reduced NO_3_^−^ uptake and transport under declining SWC can lead to changes in sap pH. Changes of sap pH of both *P. sativum* and *H. vulgare* were, however, similar in both FN and LN treatments (Figure 6), suggesting that a short-term reduction in NO_3_^−^ availability has little or no effect on sap pH. Thus, we can clearly say that our data collected from four different species do not support the model proposed by Lips (1997).

#### Consequences of Short-Term Low Nitrogen Treatment Under Drought

We used two species, namely *P. sativum* and *H. vulgare*, for follow-up experiments where the combined effects of short-term deficiency of both water and nitrogen were analyzed in detail. For both species, there were no short-term effects of NO_3_^−^ availability on the whole plant TR (Figure 1). This is not surprising considering that leaf NO_3_^−^ concentration was stable over the experimental time frame in *P. sativum* plants that were supplied with 0.2 mM NO_3_^−^ relative to plants supplied with 4 mM NO_3_^−^ (Figure 2). In *H. vulgare*, there was a significant decrease in NO_3_^−^, and yet leaf NO_3_^−^ concentration was eight times higher compared to *P. sativum* leaves (Figure 1). In other words, we assume that the reduction in leaf NO_3_^−^ amount in the LN treatment of plants in solid media was not strong and fast enough over the duration of the experiment. It could explain different conclusions of Chapin et al. (1988) where NO_3_^−^ content in the leaves of plants rapidly transferred to N-free solution decreased to 30–40% of control plants within 2 days. They also observed a simultaneous decrease in TR, probably as a consequence of higher ABA concentration in leaves.

In our short-term experiments, we also did not observe any interactive effects of NO_3_^−^ and water deficiency on TR. We found a transient increase of sap pH in *P. sativum*. This pattern of pH change has been previously observed in some other species (Gloser et al., 2016) but the origin and the regulatory implications for the leaf apoplast remain unclear. We therefore conclude that NO_3_^−^ availability in soil is not linked to the modulation of transpiration in the short-term (up to 2–6 days) as long as leaf NO_3_^−^ levels remain stable. Lower NO_3_^−^ levels in leaves, however, may contribute to stomatal regulation over longer periods of N-starvation as suggested by Chapin et al. (1988).

## Data Availability Statement

The raw data supporting the conclusions of this article will be made available by the authors, to any qualified researcher after request.

## Author Contributions

VG and CG conceptualized the main research questions, designed experiments and wrote the manuscript. VG, MD, DM, BP, and PG collected data. VG and MD performed the data analyses. All authors contributed to the article and approved the submitted version.

### Conflict of Interest

The authors declare that the research was conducted in the absence of any commercial or financial relationships that could be construed as a potential conflict of interest.
