# Supplementary material for: Early Changes in Nitrate Uptake and Assimilation Under Drought in Relation to Transpiration
Source: Front Plant Sci. 2020 Dec 23;11:602065. doi: 10.3389/fpls.2020.602065 (PMC7793686; doi:10.3389/fpls.2020.602065)
Supplement: Supplementary file 1 [file Data_Sheet_1.pdf]

Supplementary Material

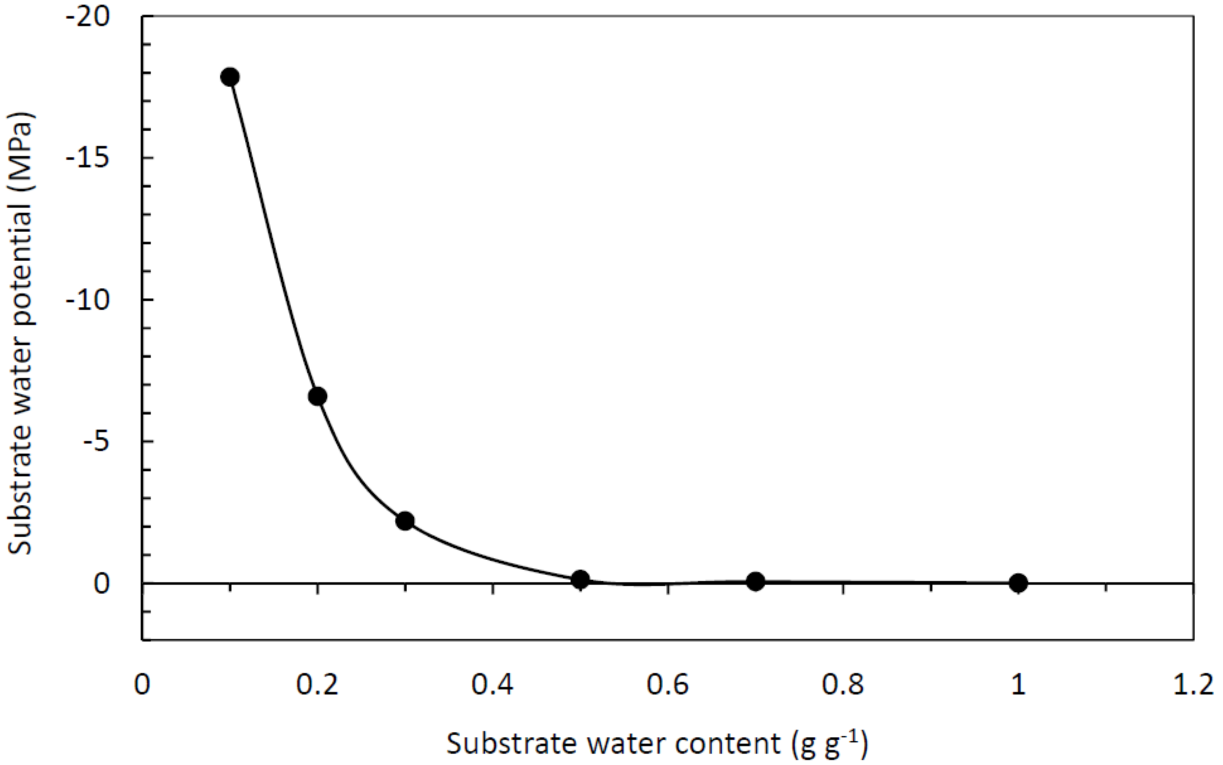

Supplementary Figure 1. The relationship between substrate water content and the water potential of the substrate used in all experiments.
